# Production Efficiency and Utility Features of Broiler Ducks Fed with Feed Thinned with Wheat Grain

**DOI:** 10.3390/ani12233427

**Published:** 2022-12-05

**Authors:** Jakub Biesek, Mirosław Banaszak, Małgorzata Grabowicz, Sebastian Wlaźlak, Marek Adamski

**Affiliations:** Department of Animal Breeding and Nutrition, Faculty of Animal Breeding and Biology, Bydgoszcz University of Science and Technology, Mazowiecka 28, 85-084 Bydgoszcz, Poland

**Keywords:** broiler duck, cereal grain, economics, meat quality, production, semi-intensive system

## Abstract

**Simple Summary:**

The production of broiler ducks on farms (incl. small-scale farms) can be carried out using various feeding and maintenance systems. During times of increasing feed prices and many challenges, a solution is sought that will result in efficient production. Owing to the high cost of complete feed, partial replacement with a wheat grain (10–40%), often from the farm’s resources, could reduce these costs. In this study, we found no adverse effects on the production performance or on the quality characteristics of meat. Moreover, an increased profit was shown according to analysis of the potential sales of duck carcasses, which is the essence of production with changes and challenges faced by producers in the agri-food market.

**Abstract:**

The aim of this study was to evaluate the production efficiency (economics), growth, and meat quality of ducks fed with feed partially replaced with wheat. A total of 200 ducks were reared for 49 days. Each group consisted of 50 ducks (5 pens with 10 birds). For slaughter, 10 birds per group were chosen. The control group (C) was provided with a complete feed. In the experimental groups, from 42 to 49 days, the feed was replaced with wheat grains at the level of 10% (W10), 20% (W20), or 40% (W40). In the W20 and W40 groups, the cost of feed was reduced. In the W40 group, the profit per 1 kg carcass was increased by PLN 3.34 (more than 24% higher than the C group profit). A higher percentage of pectoral muscles and intramuscular fat was observed in the W20 group, with lower water content. A lower water-holding capacity (WHC) was observed in the duck leg muscles in group W40. The muscles from the W20 group had higher protein, collagen, and water content, and the fat was highest in the W40 group. A lower toughness of cooked meat was observed in the W20 group, and lower shear force in the pectoral muscles of groups C and W40. Thinning feed with wheat grains could represent an alternative to conventional feeding of broiler ducks, owing to reduced feed costs, with no negative impact on utility features, including growth, except the share of pectoral muscle and water absorption traits.

## 1. Introduction

In Poland, there has been dynamic development in duck meat production in recent years. Poland is the third country in terms of production volume after France and Hungary in the European Union (67.2 thousand tons of carcass weight in 2021). Despite the SARS-CoV-2 coronavirus pandemic and avian influenza outbreaks, the production market remained stable, and an upward trend has been recorded since 2020 (70.1 thousand tons) [1]. Development of the poultry meat production sector has resulted in a need to optimize feeding methods. A balanced ratio is critical to achieve satisfactory production results and feed conversion [2]. The cost consumption of feed components, mainly cereal grains, depends on weather conditions, the economic and financial situation, the demand of other areas of industry and animal production, and petroleum prices [3]. The volatility of feed prices on the market determines the profitability of production.

Feeding costs in poultry constitute 60–70% [4], and according to Yegani and Korver [5], even up to 75% of the entire production costs. Owing to increasing prices of feed components, alternatives to conventional poultry feeding are being sought [4]. To date, research has been undertaken on the use of byproducts of the agri-food industry [6], as well as the partial replacement of a complete feed with cereal grains [7]. This activity is a response to the current challenges in the economics of poultry meat production. The practical application of such a model of poultry feeding is an opportunity for small-scale farms, where selected feed ingredients are available [4].

Wheat is a commonly used grain for the production of feed. The high starch content in wheat grains makes them a good energy source. Adding whole wheat grains is advantageous, owing to the lower price than complete feed [8]. In the available literature, wheat feeding has been used in various poultry species, including Pekin ducks. Replacing complete feed with whole wheat grain reduced the feed conversion ratio (FCR) and increased body weight (BW). The beneficial effect of wheat was also reflected in the slaughter yield and the weight of the pectoral muscles [9]. There have also been studies on the incorporation of maize in feeding Muscovy ducks. Researchers reported an effect in reducing feed costs by adding maize using the loose-mix feeding and free-choice feeding methods [10]. Similar research results were reported by Arroyo et al. [11]. Using maize and triticale in the final period of duck rearing decreased feed intake (FI), resulting in an improved economy for the producer.

The purchase of cereal grains supports local farms and reduces the negative impact of conventional nutrition on the environment [11]. Replacing complete feed with wheat at the level of 15% in the last two weeks of duck rearing influenced the physicochemical characteristics of the pectoral and leg muscles [12]. According to the authors, the changes mainly concerned the color of the pectoral muscles and the content of amino acids (threonine and valine). Changes in the content of collagen, crude fat, alanine, arginine, and proline characterized the leg muscles of ducks fed with wheat. Partial replacement of maize with triticale in feed positively influenced the nutritional quality of poultry meat protein without affecting its chemical composition otherwise [13]. By replacing wheat with triticale, a more favorable fatty acid profile was achieved in the pectoral muscles of broiler chickens [14].

Moreover, the inclusion of whole grains of triticale increased the yield of muscle and fat of Ross 308 chickens [15]. When this type of grain was used in a free-choice feeding system, no adverse effect was observed on carcass characteristics or meat quality [16]. Texture parameters are among the features that shape good-quality poultry meat. The texture properties of meat are dependent on its chemical composition, including fat and connective tissue content, as well as the thickness of muscle fibers. In addition to the physicochemical factors affecting the texture of meat, the origin, age, sex, housing system, and nutrition are essential [17]. Meat texture evaluation methods are performed using Warner-Bratzler knives and MORS analysis (Meullenet–Owens razor shear). The tenderness of meat directly affects its palatability and is one of the most important aspects for consumers [18].

Therefore, it was justified to undertake research in which the following research hypothesis was formulated: Partial replacement of complete feed with wheat grain in the last week of Cherry Valley duck rearing influences production efficiency, including the economics and growth performance, carcass characteristics, and meat quality. Modifying feed with different raw plant materials can support the local market, especially for small-scale producers. Farms have the option of selling products based on short supply chains. Among consumers, direct selling is perceived as attractive, and products from small farms are equated with higher quality and better animal welfare. It increases their competitiveness against sales by large retail chains [19].

Our research raises the topic of the current situation in the poultry market. Many challenges are caused by the need to reduce the cost of feeding poultry, particularly on small-scale farms, where local raw feed materials can be used. There are no requirements or restrictions regarding the amount of wheat used in poultry feeding, especially for ducks. It is known that feeding only with wheat grain is not suitable and would not provide the optimal amount of nutrients, but an increased proportion of up to 40% of a complete feed could be a beneficial solution. Accordingly, the aim of this study was to evaluate the production efficiency indicators, carcass characteristics, and meat quality of Cherry Valley ducks fed with feed partially replaced with wheat grain during the last week of rearing.

## 2. Materials and Methods

The experiment was carried out following the applicable regulations (Directive no. 2010/63/EU of 22 September 2010 on the protection of animals used for scientific purposes and the Act of 15 January 2015 on protecting animals used for scientific or educational purposes, item 266, Journal of Laws of the Republic of Poland).

### 2.1. Animals and Experimental Design

In this study, two hundred 1-day-old Cherry Valley broiler ducks were used. The ducks were purchased from the waterfowl hatchery (Greater Poland Voivodeship, Poland). The ducks were reared on a private farm. The farmers took care of the ducks, i.e., performed routine work in the duck house (flock service). The research team took samples and controlled the production indicators. The rearing of the ducks lasted 49 days. The ducks were divided into four equal groups. Each group was divided into five repetitions of 10 birds each. The pen order was randomized. The density per 1 m^2^ of the area was up to 17 kg of live birds. The environmental conditions aligned with the duck rearing standards (semi-intensive system). The pen size was 2 m^2^. All pens were identical and constructed of metal frames and stainless-steel mesh. The temperature in the duck house was, on average, 26 °C at the beginning of the rearing period and decreased to 20 °C (at the duckling level). Until week 4, the ducks had access to an additional heat source (30 °C). The birds were kept on cut wheat straw litter. The ducks had constant access to fresh water and feed. Nipple drinkers (2 per 10 ducks) and feeders in the backfill form (7 cm of length per duck) were provided. The feeding was divided into two periods: from the 1st to the 28th day, starter feed was used; from the 29th until the 49th day, grower or grower feed was partially replaced with wheat grain. The feed was granulated. The first group was the control group (C), which was fed a commercial starter and grower feed. The experimental groups were fed the same diet. However, on days 43–49, they received grower feed partially replaced with wheat. The share of wheat was as follows in the groups: W10, 10%; W20, 20%; and W40, 40%. The wheat feed was homogeneous. The wheat grains came from fodder crops.

There are no clear guidelines for the use of wheat in duck nutrition. Most available descriptions are for broiler chickens and laying hens. For this species, it is specified that there are no restrictions on the use of wheat feed. Similarly, triticale is described for use only in hen species. It has also been reported that its share should be 30–40% or up to 20% (broiler). Accordingly, we determined 20% as optimal proportion and assumed a lower dose (10%) and a higher dose (40%). Therefore, there is a need to research the rearing of ducks, as little is known about this species in terms of production and requirements. It should be noted that in wheat, the limiting amino acids for poultry are methionine and lysine [20].

In our country, wheat is one of the primary feed components for monogastric animals. Feed granulation was performed using an RTH-150 granulator (Pelleto, Poznań, Poland). After 49 days of rearing, two ducks were randomly selected from each replication (10 ducks in total from each group). The ducks were slaughtered after being stunned with electric current by cutting the spinal cord between the first cervical vertebra and the occipital condyle. The carcasses were plucked and gutted. The head and feet at the ankle joint were cut off. The prepared carcasses were cooled in a refrigerator (Hendi, Robakowo, Poznań, Poland) at 4 °C for 24 h and stored for further laboratory analyses of the meat quality.

### 2.2. Analytical Composition of Feeds

The feed was collected in sterile zip bags in 0.5 kg volume. The samples were analyzed in the laboratory by near-infrared transmission spectrophotometry (NIR) with calibration on artificial neural networks (ANNs). A FOSS InfraXact apparatus (FOSS, Hilleroed, Denmark) was used. The analyses were performed according to ISO standards (ISO 12099:2017) [21]. The content of dry matter, crude ash, total protein, crude fat, crude fiber, and starch was tested. After partial replacement with wheat grain, grower feed was also analyzed in comparison with the control feed.

### 2.3. Growth Performance and Production Efficiency

All ducks were weighed on the first day of rearing and each subsequent week (7 dates). The feed intake (FI) was monitored daily (feed of a known mass was poured into the feeders, and uneaten feed was weighed daily). The average FI for one duck was calculated based on the data (amount of feed intake) for the whole group. Spilled feed was not considered. However, visually, these amounts were imperceptible because the feeders were characterized by a wide and high edge, which prevented the feed from spilling out. Deaths of ducks were recorded. Weight gain was calculated based on the collected body weight (BW) and FI data (BWG=final body weight−initial body weight). The average daily feed intake ($\mathrm{ADFI}=\frac{\mathrm{total}\;\mathrm{feed}\;\mathrm{intake}\;\mathrm{per}\;\mathrm{one}\;\mathrm{duck}}{49\;\mathrm{day}}$) and feed conversion ratio ($\mathrm{FCR}=\frac{\mathrm{total}\;\mathrm{feed}\;\mathrm{intake}\;\mathrm{per}\;\mathrm{duck}}{\mathrm{BWG}}$) were calculated during the first and second feeding periods and for the entire rearing period. The ducks’ growth rate was calculated for each week ($\mathrm{GR}=\frac{\left(\mathrm{final}\;\mathrm{body}\;\mathrm{weight}-\mathrm{initial}\;\mathrm{body}\;\mathrm{weight}\right)}{0.5\;\left(\mathrm{initial}\;\mathrm{body}\;\mathrm{weight}+\mathrm{final}\;\mathrm{body}\;\mathrm{weight}\right)}\times 100$). Based on the obtained data, the European Production Efficiency Factor ($\mathrm{EPEF}=\frac{\mathrm{viability}\;\left(\%\right)\times \mathrm{BW}\;\left(\mathrm{kg}\right)}{\mathrm{age}\;\left(d\right)\times \mathrm{FCR}\;(\frac{\mathrm{kg}}{\mathrm{kg}})}\times 100$) and the European Broiler Index ($\mathrm{EBI}=\frac{\mathrm{viability}\;\left(\%\right)\times \mathrm{average}\;\mathrm{daily}\;\mathrm{gain}\;\left(\mathrm{kg}\right)}{\mathrm{FCR}\;(\frac{\mathrm{kg}}{\mathrm{kg}})}\times 100$) were also calculated.

The feed costs per duck were calculated based on the actual feed prices throughout the experiment. The gross price of the starter feed was PLN 2.06 per 1 kg, and the grower price was PLN 1.98 per 1 kg. The price of 1 kg of wheat was PLN 1.46. The feed price with 40% addition of wheat was PLN 1.77, PLN 1.88 with 20% wheat, and PLN 1.93 with 10% wheat (prices from 1–7 December 2021). The cost of feed was calculated for 1 kg of live body weight and the profit for the sale of the carcass, taking into account the feed costs, assuming the average price on the free market (e.g., direct sales system or short supply chains) of PLN 16 gross (data from 27 January 2022). Based on the 10 available sources (in person, on the Internet, and offers for local direct sales from farms in the Kuyavian-Pomeranian Voivodship in Poland), carcass sale prices were analyzed, and the average value was calculated. It was assumed that the feed cost in the control group was 100%, concerning which the percentage reduction in the price of feeding in the experimental groups was demonstrated. Based on the results, the profit of selling the entire flock was simulated, considering the deaths in the groups. The amounts were calculated based on the profit per 1 kg carcass, considering each group’s average carcass weight (the number of ducks at the end of rearing after the deduction of dead birds, was multiplied by the average carcass weight and then by the amount per kg).

### 2.4. Carcass Features and Meat Quality

Within 45 min of slaughter, the pH of the pectoral muscles was measured using a pH meter (Elmetron, Zabrze, Poland) with a dagger electrode. The device was calibrated with buffers of pH = 4.00, 6.00, and 9.00. The measurement was repeated after 24 h [22]. The carcasses were weighed (Radwag, Radom, Poland) and dissected. The following were distinguished successively: abdominal fat, neck, wings (with skin), skin with subcutaneous fat (including the skin from the neck), pectoral muscle major and minor, and trimmed leg muscles (drumstick and thigh). The leg bones and the trunk are described as carcass remains. All items were weighed.

The pectoral and leg muscles were subjected to physicochemical analyses. Using a CR-400 colorimeter (Konica Minolta, Tokyo, Japan), the color of the pectoral and leg muscles from the inside (in the place of pure muscle tissue) was analyzed. The color is presented on the CIELab scale. The L* parameter indicates lightness, a* indicates redness, and b* represents yellowness. The pectoral muscles used for drip loss (DP) analysis were weighed and then placed in zip bags (so that the lost water would drain into a larger bag) in a 4 °C refrigerator for 24 h. After the test time had expired, samples were weighed again. The drip loss was calculated according to the difference in sample weight. The water-holding capacity (WHC) of pectoral and leg muscles was determined in ground samples. The muscles were ground in a meat grinder (Hendi, Poznań, Poland). The samples weighing 0.300 g (±0.005 g) were weighed and placed on Whatman filter papers. Prepared samples were placed under a weight of 2 kg for 5 min. Then, the trials were weighed out again. The result was obtained the same way as for drip loss ($\mathrm{DP}\;\mathrm{or}\;\mathrm{WHC}=(100-\frac{\mathrm{the}\;\mathrm{initial}\;\mathrm{weight}\;\mathrm{of}\;\mathrm{muscle}}{\mathrm{final}\;\mathrm{weight}\;\mathrm{of}\;\mathrm{muscle}})\times 100$). For the chemical composition analysis, ground samples of pectoral and leg muscles with a total weight (in the group) of 90 g were used. The analyses were performed with a FoodScan apparatus (FOSS, Hilleroed, Denmark) with near-infrared transmission (NIT) spectrophotometry. The methods of testing the characteristics of carcasses and meat quality are described in the study by Banaszak et al. [23].

The pectoral muscles were subjected to texture analysis using a TA.XT plus C device (Stable Micro Systems, Cereus Wena, Toruń, Poland) using prepared samples of raw and cooked meat. The pectoral muscles were prepared as 1 × 1 cm samples. The heat treatment tests were carried out in a W410E water bath (Labo Play, Bytom, Poland) at 80 °C for 40 min. The Warner–Bratzler test was performed using a flat knife (the test speed was 1.50 mm/s). The samples were placed on a heavy-duty platform. The test results are presented as meat firmness (N) and toughness (N × s). Using Volodkevich jaw grips, the firmness of the cooked pectoral muscle samples was tested (test speed was 2.00 mm/s), and using the Meullenet–Owens razor shear (MORS) attachment, the shear force (N) of the pectoral muscles placed on the table was analyzed (four measuring points per sample (average value); the test speed was 1.50 mm/s). A TA.XT plus C device was used with a 50 kg load cell. Muscle texture studies were performed according to Gornowicz et al. [17] and Guzman et al. [24].

### 2.5. Statistical Calculation

The numerical data were analyzed in the statistical program Statistica 13.3. (Tibco, Statsoft, Kraków, Poland). The production results (growth, feed factors, and economics) were analyzed in 5 replicates per group. Based on production results, including growth, feed indicators, and efficiency, mean values from all birds were calculated for each group (5 repetitions per group). On the other hand, verification of laboratory results (meat features and feed composition) was based on 10 repetitions for each group (1 duck was an experimental unit (replication)). Feed was taken from each pen (5 samples per group). Two replicates (a total of 10) were performed from each trial. The mean values of the quantitative traits for each studied group (C, W10, W20, and W40) and the standard error of the mean (SEM) were calculated. The standard deviation (±SD) was calculated for each examined feature within the groups. Calculations were performed by selecting the option of one-dimensional results. A one-way analysis of variance was used. The normal distribution of the dependent variable results in each analyzed group was verified by a Shapiro–Wilk test (*p* < 0.05). The homogeneity of variance was checked using an additional Levene’s test (*p* < 0.05). The analyzed dependent variables were subjected to Tukey’s post hoc test (multiple comparisons of statistically significant differences) with *p* < 0.05.

## 3. Results

### 3.1. Analytical Composition of Feeds

Table 1 shows the analytical results of the chemical composition of the feeds used in the experiment. The starter feed contained ingredients appropriate broiler duck growth (based on the feed manufacturer’s declaration and label). Significant differences were observed in the grower feed between the control and experimental groups, apart from the dry weight (*p* = 0.760). A statistically significantly higher content of ash and protein was found in the feed of groups C and W10 than in that of groups W40 and W20 (*p* < 0.001). The fat and fiber content in the feed from group C was significantly higher than that in the W40, W20, and W10 groups (*p* < 0.001). Additionally, the feed of the W40 group was characterized by lower fat and fiber content than that of the W20 and W10 groups. The starch content was significantly higher in the W40 group and decreased successively in the W20, W10, and C groups (*p* < 0.001).

### 3.2. Growth Performance and Production Efficiency

There were no statistically significant differences in body weight, weight gain, or average daily feed intake and its conversion ratio between the groups (*p* > 0.05). The production results of ducks, irrespective of the feed supplied, were similar. The growth rate of the ducks was similar in all groups (Table 2).

Statistically significantly reduced feed costs per duck were observed during the feeding period with grower feed (29–49 days) and in the entire rearing period (1–49 days) in groups W40 and W20 compared to the control group (*p* = 0.007; *p* = 0.019, consecutively). The percentage value of the reduced costs showed significant differences between the W40 group and the C group (*p* = 0.044). Costs were cut by more than 9%. In the W20 and W10 groups, the reduction in feed costs relative to the prices in the control group was substantial (more than 8–7%). Calculations of profit (taking into account feed costs) per 1 kg of carcass weight showed a significantly higher value in group W40 than in group C (*p* = 0.033) by PLN 3.34 (Table 3), assuming that in each group, the flock was 50 ducks and the final flock density was 45 (C), 49 (W10 and W20), and 48 (W40). The quantitative difference ranges from PLN 298.82 to PLN 555.93 (an increase of 19–37% in profit) compared to the control group (C).

### 3.3. Carcass Features and Meat Quality

Table 4 shows that feeding with feed replaced with 40% wheat statistically significantly decreased the percentage of pectoral muscles in the carcass in the W40 group compared to the C group (1.66%; *p* = 0.031). With respect to other features (leg muscles and fatness), no statistically significant differences were observed between the groups (*p* > 0.05).

Table 5 presents the results of the physicochemical features of the pectoral and leg muscles. A statistically significantly higher pH of the pectoral muscles was demonstrated 45 min after slaughter in the W20 group compared to the C group (*p* = 0.025). The feed replaced with 40% wheat resulted in a significantly increased value of WHC in the pectoral muscle in the W40 group compared to the W10 group. The lowest WHC value was found in the C group compared to the experimental groups (WHC expressed as a percentage of lost water) (*p* < 0.001). However, in the case of leg muscles, only the W40 group showed a statistically significantly lower WHC value than the C group (*p* = 0.017).

All groups were characterized by significantly different protein content (in ascending order): group C < W20 < W40 < W10 (*p* < 0.001). The fat content was significantly higher in the W20 group compared to the W10 and C groups. Furthermore, group C differed (less fat) from the W40 group (*p* < 0.001). A higher water content characterized pectoral muscles in group C relative to groups W20 and W10 (*p* < 0.001). Leg muscles from group W20 were characterized by significantly higher protein and collagen content (*p* < 0.001 and *p* = 0.004) relative to groups C and W40. Moreover, group W10 had a higher protein content than group W40. Fat content in leg muscles was higher in group W40 compared to groups C and W20 and higher in group W10 than group W20 (*p* < 0.001). The leg muscles in the W20 group were characterized by a higher water content than the W10 and W40 groups. Additionally, significantly higher water content was found in the C group compared to the W40 group (*p* < 0.001).

The results regarding the texture of raw and cooked pectoral muscles are also shown in Table 5. Cooked muscle texture was significantly higher in the W10 group compared to the W20 group according to Warner–Bratzler test results (*p* = 0.036 and *p* = 0.028, respectively). MORS analysis showed that the shear force in the W20 and W10 groups was statistically significantly higher than that in the W40 and C groups (*p* < 0.001).

## 4. Discussion

This study was performed during the production of broiler ducks on a small-scale farm. It was a pilot activity in terms of the analyses performed. The follow-up could be extended to detailed feed research, including metabolic energy, nutrient digestibility, and market analysis.

### 4.1. Feed Composition, Growth, and Production Efficiency

The analytical results were similar to those declared by the producers of complete feed (footnotes in Table 1). Although the protein content in the feed in the W20 and W40 groups was significantly lower, it did not negatively impact the production results and the tissue composition of the carcasses. In our research, no adverse effect of partial feed replacement with wheat grain in the last week of rearing ducks was observed on body weight (BW), body weight gain (BWG), average daily feed intake (ADFI), or feed conversion ratio (FCR). Similar results were reported by Kokoszyński et al. [9]. Partial replacement of feed with wheat at the level of 5% in the fourth week of rearing, which was increased to 20 and 30% in the seventh week of rearing, did not affect the final BW, FI, or FCR. The use of whole wheat grains (15%) in the last two weeks of rearing also did not affect the production results of Pekin ducks [12]. In a study on adding high-moisture maize at a concentration of between 5 and 10% between the 29th and 50th days of rearing, alternative feeding did not affect the final BW, BWG, FI, or FCR of Pekin ducks [25]. At the beginning of life, ducks are characterized by dynamic weight gain, so they use the initially provided complete feed [26]. The subsequent dilution of the feed with wheat grain did not affect the weight gain, owing to the reduced growth intensity in the last week of rearing. Xie et al. [27] concluded that appropriate supplementation based on an easily digestible amino acid profile allows Pekin ducks to be fed a low-protein diet (as low as 15% crude protein) without adversely affecting the growth performance and efficiency of the carcass. Similar conclusions were described by Baeza and Leclercq [28] when conducting research using Muscovy ducks. According to Wen et al. [29], lysine plays a significant role in protein synthesis, and its deficiency may reduce growth, feed consumption, and slaughter yield. The feeding recommendations for the ducks indicate that the protein level in the feed can be between 16 and 18%, so the conception of the above ratios of wheat is in line with the protein content recommendations [30]. For example, after the 4th week of duck rearing, the total protein may be 14–15% per 1 kg of feed (88% of dry matter). Mohanty et al. [31] found that 16% protein in duck feed was adequate. In our research, the lowest protein level was in the W40 group (16.29%). We did not analyze the composition of amino acids, which should be investigated in future studies.

Duck deaths were recorded in all groups as a result of weak ducklings. According to our results, feed costs per bird were lower in the group fed with feed wheat replaced at 20 and 40% levels compared to the control group fed with standard feed. The experimental feed costs decreased the most in the W10, W20, and W40 groups by 7.21%, 8.10%, and 9.24%, respectively, compared to complete feed group. Feed costs can be reduced in poultry nutrition using barley and triticale. According to Pogosyan et al. [7], adding these cereal grains to the diet of broiler chickens reduced the feed costs per kg of carcass production by 2.8–3.9%. Using a high-wheat feed reduced the feed cost and the total cost of production of chickens from USD 1.019 to USD 0.972 [32]. Raising Muscovy ducks in cornfields with grain-based nutrition can reduce producers’ costs. This system increased the profit per duck by 22.8% compared to keeping birds according to the conventional method. Furthermore, such a maintenance system allows for improved land development for plant production and is conducive to obtaining increased profits from the cultivation of cereals [33]. An alternative to standard duck feeding methods is the addition of dried corn stillage (DDGS). According to Kowalczyk et al. [34], using the feed containing 25% DDGS to feed reduced the purchasing cost per ton. In a study by Diarra et al. [35], selected indicators of the effectiveness of Cobb chickens were analyzed using cassava root meal and copra meal. The authors reported that this procedure significantly reduced feed costs by USD 0.74. Moreover, a reduction of as much as USD 0.9 in feed costs per 1 kg of body weight of chickens achieved. Increased profitability of the production of broilers was also reported with the use of cassava waste meal (CWM) at the level of 10% (significantly reduced cost of 1 kg of feed and cost/weight gain) [36]. An alternative method of feeding ducks is the use of 15% giant Salvinia (*Salvinia molesta*, a floating plant that occurs naturally in South America and Asia). In addition to increasing the income relative to feed cost, birds were found to have increased body weights and feed conversion rates in this experimental group [37]. Attention should also be paid to other factors that significantly affect the profitability and efficiency of production, such as the maintenance system [38], stocking density per 1 m^2^ of surface area [39], and the use of organic acids or phytobiotics [40].

### 4.2. Carcass Features and Meat Quality

In our research, ducks fed with feed replaced with 20% wheat showed a higher percentage of pectoral muscles in the carcass than in the W40 group. Kokoszyński et al. [9] found that partial replacement of complete feed with wheat grain from week 4 of rearing increased the percentage of pectoral muscles in the carcass.

A reduction in the pH value (acidification) of the pectoral and leg muscles results from biochemical changes in the muscle cells. Glycolysis (glucose breakdown) and its byproduct, lactic acid, are essential in shaping this feature [41]. According to Qamar et al. [42], the pH of the pectoral muscles depends on physiological changes and the nutrition of ducks. The use of maize after a prolonged storage period (4 years) at the level of 100% reduced the pH value of the pectoral muscles compared to the group with a 50% addition of maize. The literature shows the influence of nutrition on the pH of meat. Increased pH was observed only in the group of ducks fed feed diluted with 20% wheat. It can also be assumed that pH was also influenced by short-term factors and various individual factors (for example, way of bleeding and level of stress) related to the peri- and postmortem periods. The literature reports that the intramuscular fat content and muscle pH are associated with texture [43]. In our research, in group W20, the pH and fat content were increased.

The worst water-holding capacity (high WHC value) was characteristic of the pectoral muscles of ducks from the W40 group. In the case of leg muscles in this group, a better WHC (higher water retention capacity) was found compared to the control group. WHC is a crucial parameter in assessing meat quality, affecting its tenderness and juiciness. The WHC value is related to the release of water from intercellular spaces [44] and its permeability through cell membranes [45]. It also depends on the degree of protein denaturation caused by reduced pH [46]. The use of alternative protein sources to soybean meal (SBM) in the form of yellow lupine, narrow-leaved lupine, rapeseed meal, and pea did not affect the WHC of Pekin duck muscles [47].

In a study by Laudadio and Tufarelli [48], an increase in WHC was reported in the pectoral muscles of chickens fed micronized peas (*p* < 0.01). In a study by Banaszak et al. [23], the replacement of SBM with yellow lupine increased the WHC of the leg muscles of Cherry Valley ducks (*p* = 0.019). Other authors also reported a relationship between changes in the color of meat (increase in a* vs. L* and b* values) with antioxidant properties and the ability to maintain water in meat [49]. However, in our research, no significant differences in the meat color parameters were observed between the groups.

The pectoral muscles of ducks from the W10 group were characterized by increased protein content, and increased intramuscular fat was found in the W20 group. The water content in the pectoral muscles of ducks fed with grower feed supplemented with 20% wheat was the lowest. Intramuscular fat (ITF) is defined as a flavor carrier and contributes to the tenderness and juiciness of meat. Muscle ITF content may depend on the composition of the feed and is related to the concentration of energy and fat in the feed [50]. According to the authors, reducing the amount of the nutrients mentioned above in the feed by 10% increased the water content (*p* ≤ 0.05) and ITF (*p* ≤ 0.05) of Ross 308 broiler chickens. Furthermore, an increase in protein content (*p* ≤ 0.05) in the muscles was reported in the group with reduced energy and protein content (10%) and the control group. Our research showed increased protein, collagen, and water content in the leg muscles of ducks from the group in which feed was replaced with 20% wheat. Increased ITF content was found in the W40 group. Moreover, the crude fat content in the feed was the lowest in this group, which does not confirm the relationship between feed fat content and muscle ITF. Other results were reported by Kokoszyński et al. [12]. Partial replacement of the complete feed (15%) with whole wheat grain reduced the fat (*p* = 0.026) and collagen (*p* = 0.029) contents in Pekin duck leg muscles. According to Infante-Rodriguez et al. [51], using feed with varied energy content does not affect the chemical composition of the leg muscles of broiler chickens. The age of the ducks affects the chemical composition of the pectoral and leg muscles. As a feed energy source, vegetable oils did not affect the collagen, fat, or water content but increased the protein content of duck meat (*p* < 0.05) [52]. Research conducted on Pekin ducks showed that the muscles of birds slaughtered in the 9th week of rearing were characterized by higher protein content and lower ITF and water contents compared to ducks slaughter in the 7th or 8th weeks of rearing [53].

The cooked pectoral muscles of ducks from group W20 were characterized by the lowest force necessary to cut the sample (*p* = 0.036). The tenderness of the meat is related to the content of water and connective tissue (collagen) in the meat. Water fills the intercellular spaces, positively affecting the parameters of the meat texture [44]. Another factor influencing toughness is muscle protein complex (actomyosin) breakdown, which occurs under high temperatures [54]. In our research, more tender meat contained less water and more fat. According to Larzul et al. [55], meat texture properties may depend on the bird’s genotype. Cooked Pekin duck pectoral muscles were characterized by a minor force required to cut, which confirms that it is more delicate than Muscovy duck meat. According to Park et al. [56], using oregano in powder form as a bioactive substance does not affect the cutting power of cooked Cherry Valley duck meat. MORS analysis revealed that the most favorable shear force occurred in the pectoral muscles of ducks from groups C and W40. In a study by Feye et al. [57], the addition of a mixture of formic acid and sodium formate (0.25 and 0.5%) reduced the shear force (*p* < 0.001) compared to the group fed with the addition of formalin in the forage. MORS analysis is also widely believed to be an effective instrumental method for assessing muscle myopathy, e.g., wooden pectoral muscle [58].

## 5. Conclusions

Our preliminary study showed that dilution of feed with wheat grain (10, 20, and 40%) in the 7th week of rearing broiler ducks did not negatively affect the production results (growth). The body weight and weight gain did not differ significantly. The results with respect to meat quality are manageable and the most favorable (except for the share of pectoral muscles and water absorption) with 40% wheat. Owing to the dynamic increase in feed costs, wheat may be an alternative to less available and more expensive feed components. With the aim of production efficiency (economics), dilution of feed with wheat for ducks is recommended at the level of 20–40%. With increasing feed prices, this form of feeding could be used, especially given that the data indicate an increase in profit from the sale of carcasses by about PLN 300–500 per group compared to control feeding (19–37%), in line with the trends of local direct sales, mainly for small-scale farms. Further research should be conducted on feed digestibility and the feed–digestion relationship.

## Figures and Tables

**Table 1:** Analytical composition of grower feed with and without wheat grain addition and starter feed.

Item	Grower Feed and Feeds with Wheat Grains ^1^				SEM ^3^	*p*-Value ^4^	Starter Feed for All Groups ^2b^
	C ^2a^	W10	W20	W40			
Dry matter (%)	87.24 ± 0.13	87.17 ± 0.09	87.24 ± 0.05	87.39 ± 0.93	0.07	0.760	87.44 ± 0.12
Crude ash (%)	4.76 ^a^ ± 0.02	4.75 ^a^ ± 0.03	4.69 ^b^ ± 0.04	4.68 ^b^ ± 0.03	0.01	<0.001	5.01 ± 0.11
Crude protein (%)	17.22 ^a^ ± 0.15	17.28 ^a^ ± 0.13	16.71 ^b^ ± 0.15	16.29 ^c^ ± 0.14	0.07	<0.001	19.01 ± 0.09
Crude fat (%)	3.53 ^a^ ± 0.08	3.39 ^b^ ± 0.10	3.33 ^b^ ± 0.09	3.00 ^c^ ± 0.07	0.03	<0.001	3.63 ± 0.12
Crude fiber (%)	3.92 ^a^ ± 0.10	3.58 ^b^ ± 0.09	3.54 ^b^ ± 0.14	3.15 ^c^ ± 0.09	0.05	<0.001	3.94 ± 0.08
Starch (%)	42.37 ^d^ ± 0.38	43.64 ^c^ ± 0.34	44.91 ^b^ ± 0.51	46.72 ^a^ ± 0.38	0.27	<0.001	39.36 ± 0.29

^1^ Groups: C = control group; W10 = ducks fed with 10% wheat grains in feed during last week of rearing; W20 = ducks fed with 20% wheat grains in feed during 7th week of rearing; W40 = ducks fed with 40% wheat grains in feed during last week of rearing. Starter feed was given on days 1–28 and grower feed was provided on days 29–49, taking into account that on days 43–49, ducks in groups W40, W20, and W10 received grower feed with wheat grains; ^2a^, the basic grower feed was commercial. In the experimental groups, feed was partially replaced with wheat grain. The chemical composition presented in the table is analytical. The grower feed was commercial and approved for sale and feeding to ducks. According to the label, the feed contained: 17.1% crude protein, 3.7% crude oils and fats, 4.5% crude fiber, 0.87% lysine, 0.37% methionine, 0.61% threonine, 0.81% calcium, 0.66% total phosphorus, 0.16% sodium, 10,000 units vitamin A, 3000 units vitamin D3, and 25 units vitamin E; ^2b^, the basic starter feed was commercial. The starter feed was commercial and approved for sale and feeding to ducks. The chemical composition presented in the table is analytical. According to the label, the feed contained: 19.5% crude protein, 3.9% crude oils and fats, 4.2% crude fiber, 0.93% lysine, 0.42% methionine, 0.72% threonine, 0.85% calcium, 0.69% total phosphorus, 0.17% sodium, 10,000 units vitamin A, 3000 units vitamin D3, and 25 units vitamin E; ^3^ SEM, standard error of the mean; ^4, a, b, c, d^ values within a row with different superscripts differ significantly at *p* < 0.05; ±SD, standard deviation.

**Table 2:** Growth performance of broiler ducks during 7 weeks of rearing.

Item ^2^	Group ^1^				SEM ^3^	*p*-Value ^4^
	C	W10	W20	W40		
BW (g)						
1st day	49.40 ± 1.01	49.38 ± 0.85	49.96 ± 0.97	49.76 ± 1.12	0.21	0.750
28th day	1993.31 ± 100.56	1970.81 ± 117.93	1939.91 ± 198.14	1999.56 ± 156.39	30.86	0.918
49th day	3462.03 ± 276.97	3365.51 ± 214.57	3340.57 ± 151.33	3459.99 ± 168.70	44.61	0.715
BWG (g)						
1–28 days	1943.91 ± 101.47	1921.43 ± 117.89	1889.95 ± 198.33	1949.80 ± 155.83	30.88	0.917
29–49 days	1468.72 ± 237.07	1394.70 ± 168.79	1400.66 ± 106.81	1460.43 ± 53.71	33.19	0.821
1–49 days	3412.63 ± 277.67	3316.13 ± 215.17	3290.61 ± 151.34	3410.23 ± 168.44	44.68	0.715
ADFI (g)						
1–28 days	144.19 ± 7.59	137.19 ± 5.77	137.51 ± 5.42	137.92 ± 5.39	1.42	0.254
29–49 days	370.44 ± 23.99	344.78 ± 15.08	345.14 ± 16.71	351.36 ± 19.77	4.61	0.159
1–49 days	242.87 ± 13.48	226.16 ± 9.69	226.50 ± 10.22	231.10 ± 12.41	2.83	0.118
FCR (kg/kg)						
1–28 days	2.08 ± 0.13	2.01 ± 0.15	2.05 ± 0.22	2.00 ± 0.24	0.04	0.877
29–49 days	5.42 ± 0.57	5.26 ± 0.52	5.19 ± 0.46	5.06 ± 0.37	0.14	0.856
1–49 days	3.51 ± 1.01	3.36 ± 0.75	3.38 ± 0.35	3.33 ± 0.30	0.07	0.806
Growth rate (%) every week						
wk 1	120.61 ± 8.33	121.13 ± 3.30	120.43 ± 1.29	122.06 ± 2.89	0.99	0.948
wk 2	105.97 ± 3.34	104.24 ± 2.89	104.74 ± 1.32	104.81 ± 1.66	0.52	0.722
wk 3	65.65 ± 5.51	67.25 ± 1.02	67.50 ± 2.56	63.62 ± 6.05	0.95	0.482
wk 4	42.78 ± 3.20	41.82 ± 3.70	39.06 ± 9.88	44.09 ± 2.93	1.24	0.567
wk 5	18.20 ± 7.25	21.37 ± 8.08	20.46 ± 6.12	21.33 ± 4.47	1.40	0.860
wk 6	26.30 ± 4.95	22.64 ± 8.32	22.12 ± 2.08	23.26 ± 2.83	1.12	0.588
wk 7	10.30 ± 3.55	9.22 ± 3.88	11.91 ± 3.59	10.19 ± 1.52	0.71	0.638

^1^ Groups: C = control group; W10 = ducks fed with 10% wheat grains in feed during last week of rearing; W20 = ducks fed with 20% wheat grains in feed during 7th week of rearing; W40 = ducks fed with 40% wheat grains in feed during last week of rearing; ^2^ item: BW = body weight; BWG = body weight gain; ADFI, average daily feed intake; FCR, feed conversion ratio; ^3^ SEM, standard error of the mean; ^4^ no statistical differences between groups were found at *p* > 0.05; ±SD, standard deviation.

**Table 3:** Production efficiency of broiler ducks during 7 weeks of rearing.

Item ^2^	Group ^1^				SEM ^3^	*p*-Value ^4^
	C	W10	W20	W40		
Efficiency of broiler duck production						
Viability (%)	90.00 ± 7.07	98.00 ± 4.47	98.00 ± 4.47	96.00 ± 5.48	1.35	0.107
EPEF	183.24 ± 46.86	200.48 ± 35.81	195.59 ± 19.99	203.71 ± 37.53	9.67	0.818
EBI	185.86 ± 47.31	203.44 ± 42.15	198.55 ± 23.99	206.68 ± 37.99	7.75	0.821
Feed costs per duck (PLN, gross)						
1–28 days	8.32 ± 0.44	7.91 ± 0.33	7.93 ± 0.31	7.96 ± 0.31	0.08	0.255
29–49 days	15.40 ^a^ ± 0.99	14.07 ^ab^ ± 0.61	13.81 ^b^ ± 0.67	13.51 ^b^ ± 0.76	0.23	0.007
1–49 days	23.72 ^a^ ± 1.36	21.98 ^ab^ ± 0.94	21.74 ^b^ ± 0.98	21.47 ^b^ ± 0.94	0.30	0.018
Feed costs: kg of live weight (PLN, gross)	6.99 ± 0.75	6.66 ± 0.63	6.61 ± 0.32	6.32 ± 0.58	0.13	0.380
Experimental feed costs: control feeding (%)	100.00 ^a^ ± 0.00	92.79 ^ab^ ± 3.86	91.90 ^ab^ ± 6.34	90.76 ^b^ ± 6.93	1.33	0.044
Profit per 1 kg of carcass (PLN, gross)	13.46 ^b^ ± 2.18	15.13 ^ab^ ± 2.48	15.00 ^ab^ ± 1.90	16.80 ^a^ ± 2.88	0.41	0.033
Profit per flock (group, PLN, gross)	1501.41	1834.36	1800.23	2057.32	-	-

^1^ Groups: C = control group; W10 = ducks fed with 10% wheat grains in feed during last week of rearing; W20 = ducks fed with 20% wheat grains in feed during 7th week of rearing; W40 = ducks fed with 40% wheat grains in feed during last week of rearing; ^2^ item: EPEF, European Production Efficiency Factor; EBI, European Broiler Index; Experimental feed costs: control feeding was calculated as 100%. Profit per 1 kg of carcass on the free market was calculated based on the average prices for duck carcasses on the free marker (16 PLN gross, date: 26.01.2022). The calculation included feed costs, profit per flock based on the average carcass weight, flock density, viability, and profit per 1 kg carcass. ^3^ SEM, standard error of the mean; ^4, a, b^ values within a row with different superscripts differ significantly at *p* < 0.05; ±SD, standard deviation.

**Table 4:** Carcass features of broiler ducks after 7 weeks of rearing.

Item	Group ^1^				SEM ^2^	*p*-Value ^3^
	C	W10	W20	W40		
Preslaughter body weight (g)	3522.70 ± 264.91	3442.70 ± 235.48	3433.10 ± 169.38	3585.10 ± 220.49	35.63	0.397
Carcass weight (g)	2478.80 ± 145.41	2474.28 ± 165.35	2449.29 ± 126.85	2551.26 ± 192.29	24.96	0.525
Dressing percentage (%)	70.46 ± 1.96	71.89 ± 1.50	71.34 ± 0.81	71.15 ± 2.93	0.31	0.3056
Neck (g)	190.48 ± 28.52	190.88 ± 28.63	185.25 ± 20.56	187.54 ± 36.54	4.44	0.969
Neck (%)	7.71 ± 1.25	7.30 ± 1.11	7.59 ± 0.98	7.41 ± 1.59	0.19	0.937
Wings (g)	299.91 ± 20.22	298.37 ± 19.40	297.75 ± 26.39	305.99 ± 21.07	3.37	0.829
Wings (%)	12.12 ± 0.83	12.08 ± 0.70	12.16 ± 0.85	12.03 ± 0.79	0.12	0.985
Carcass remains (g)	638.45 ± 74.58	636.12 ± 151.88	621.68 ± 56.54	687.07 ± 135.27	17.46	0.590
Carcass remains (%)	25.74 ± 2.54	25.70 ± 6.24	25.41 ± 2.31	26.84 ± 4.04	0.63	0.871
Pectoral muscle (g)	539.77 ± 31.14	532.12 ± 41.16	536.99 ± 51.46	513.59 ± 57.99	7.26	0.594
Pectoral muscle (%)	21.80 ^ab^ ± 1.12	21.51 ^ab^ ± 1.01	21.91 ^a^ ± 1.60	20.14 ^b^ ± 1.80	0.24	0.031
Leg muscle (g)	307.65 ± 36.24	317.73 ± 44.15	309.88 ± 47.51	294.04 ± 51.21	6.99	0.700
Leg muscle (%)	12.42 ± 1.31	12.85 ± 1.66	12.65 ± 1.86	11.58 ± 2.06	0.28	0.389
Total muscle (g)	847.42 ± 63.35	849.85 ± 67.01	846.87 ± 82.86	807.63 ± 99.33	12.39	0.588
Total muscle	34.22 ± 2.21	34.36 ± 1.79	34.56 ± 2.75	31.72 ± 3.60	0.45	0.074
Skin with subcutaneous fat (incl. neck skin) (g)	481.30 ± 102.35	478.61 ± 129.98	475.58 ± 73.58	540.88 ± 98.07	16.23	0.439
Skin with subcutaneous fat (incl. neck skin) (%)	19.35 ± 3.53	19.31 ± 5.39	19.38 ± 2.49	21.14 ± 3.07	0.59	0.643
Abdominal fat (g)	21.24 ± 7.25	20.45 ± 10.20	22.16 ± 6.98	22.15 ± 8.18	1.26	0.960
Abdominal fat (%)	0.85 ± 0.27	0.81 ± 0.37	0.90 ± 0.25	0.86 ± 0.30	0.05	0.940
Total fat (g)	502.54 ± 107.83	499.06 ± 134.22	497.74 ± 76.61	563.03 ± 103.75	16.92	0.467
Total fat (%)	20.20 ± 3.72	20.13 ± 5.50	20.28 ± 2.56	22.01 ± 3.28	0.61	0.664

^1^ Groups: C = control group; W10 = ducks fed with 10% wheat grains in feed during last week of rearing; W20 = ducks fed with 20% wheat grains in feed during last week of rearing; W40 = ducks fed with 40% wheat grains in feed during 7th week of rearing; ^2^ SEM, standard error of the mean; ^3, a, b^ values within a row with different superscripts differ significantly at *p* < 0.05; ±SD, standard deviation.

**Table 5:** Physicochemical features of broiler duck pectoral muscle after 7 weeks of rearing.

Item ^2^		Group ^1^				SEM ^3^	*p*-Value ^4^
		C	W10	W20	W40		
Pectoral muscle							
pH 45 min		5.92 ^b^ ± 0.11	5.94 ^ab^ ± 0.06	6.04 ^a^ ± 0.09	6.00 ^ab^ ± 0.09	0.02	0.025
pH 24 h		5.73 ± 0.11	5.51 ± 0.28	5.65 ± 0.18	5.75 ± 0.19	0.03	0.051
Color							
L*		36.11 ± 3.33	36.12 ± 3.23	36.80 ± 1.85	35.79 ± 1.78	0.41	0.854
a*		16.56 ± 2.64	16.68 ± 2.21	16.96 ± 1.76	17.21 ± 1.46	0.32	0.897
b*		2.62 ± 1.34	3.18 ± 1.25	3.28 ± 0.77	2.16 ± 0.93	0.18	0.095
Drip loss (%)		1.70 ± 0.57	2.00 ± 1.02	2.43 ± 1.14	1.77 ± 0.90	0.15	0.305
WHC (%)		31.19 ^c^ ± 1.36	34.96 ^b^ ± 1.76	36.68 ^ab^ ± 2.69	39.31 ^a^ ± 2.68	0.58	<0.001
Protein (%)		20.91 ^d^ ± 0.05	21.46 ^a^ ± 0.06	21.16 ^c^ ± 0.05	21.33 ^b^ ± 0.05	0.03	<0.001
Collagen (%)		1.17 ± 0.13	1.23 ± 0.08	0.95 ± 0.38	1.12 ± 0.18	0.04	0.184
Intramuscular fat (%)		2.23 ^c^ ± 0.02	2.30 ^bc^ ± 0.02	2.71 ^a^ ± 0.03	2.33 ^ab^ ± 0.04	0.03	<0.001
Water (%)		76.90 ^a^ ± 0.13	76.52 ^bc^ ± 0.06	76.48 ^c^ ± 0.04	76.66 ^ab^ ± 0.12	0.03	<0.001
The texture of the pectoral muscle							
Flat knife (Warner–Bratzler test)							
Raw	Firmness (N)	40.57 ± 16.78	39.38 ± 12.52	40.74 ± 13.22	41.78 ± 8.27	1.99	0.982
	Toughness (N × s)	276.41 ± 90.55	268.13 ± 82.52	262.59 ± 72.18	284.77 ± 48.44	11.49	0.918
Cooked	Firmness (N)	33.81 ^ab^ ± 12.12	38.57 ^a^ ± 9.71	25.83 ^b^ ± 8.88	28.24 ^ab^ ± 9.59	1.73	0.036
	Toughness (N × s)	213.69 ^ab^ ± 66.48	254.64 ^a^ ± 56.37	178.65 ^b^ ± 51.54	197.34 ^ab^ ± 44.90	9.54	0.028
Volodkevich jaw grips (cooked meat)							
Firmness (N)		9.60 ± 4.69	10.16 ± 3.48	10.22 ± 4.03	9.06 ± 3.69	0.61	0.909
Meullenet–Owens razor shear (MORS) (raw meat)							
Shear force (N)		4.15 ^b^ ± 0.99	5.96 ^a^ ± 1.58	6.64 ^a^ ± 2.14	4.85 ^b^ ± 1.42	0.15	<0.001
Leg muscle							
Color							
L*		35.35 ± 2.72	34.96 ± 2.97	34.65 ± 2.54	34.06 ± 2.59	0.43	0.768
a*		15.10 ± 1.91	15.74 ± 3.88	13.80 ± 2.56	14.70 ± 2.55	0.44	0.488
b*		2.48 ± 0.99	3.55 ± 1.34	2.88 ± 0.94	2.38 ± 1.19	0.19	0.102
WHC (%)		42.02 ^a^ ± 8.13	37.45 ^ab^ ± 5.13	38.48 ^ab^ ± 3.55	34.00 ^b^ ± 2.59	0.92	0.017
Protein (%)		18.64 ^bc^ ± 0.05	18.80 ^ab^ ± 0.02	18.99 ^a^ ± 0.08	18.36 ^c^ ± 0.05	0.04	<0.001
Collagen (%)		1.42 ^b^ ± 0.14	1.53 ^ab^ ± 0.09	1.68 ^a^ ± 0.19	1.50 ^b^ ± 0.17	0.03	0.004
Intramuscular fat (%)		5.34 ^bc^ ± 0.01	5.84 ^ab^ ± 0.04	4.67 ^c^ ± 0.07	6.56 ^a^ ± 0.04	0.11	<0.001
Water (%)		74.88 ^ab^ ± 0.04	74.23 ^bc^ ± 0.05	75.96 ^a^ ± 0.28	73.92 ^c^ ± 0.16	0.13	<0.001

^1^ Groups: C = control group; W10 = ducks fed with 10% wheat grains in feed during last week of rearing; W20 = ducks fed with 20% wheat grains in feed during 7th week of rearing; W40 = ducks fed with 40% wheat grains in feed during last week of rearing; ^2^ Item: L*—lightness; a*, redness; b*, yellowness; WHC, water-holding capacity; ^3^ SEM, standard error of the mean; ^4, a, b, c^ values within a row with different superscripts differ significantly at *p* < 0.05; ±SD, standard deviation.

## Data Availability

Not applicable.
